# The Triple Mechanisms of Atenolol Adsorption on Ca-Montmorillonite: Implication in Pharmaceutical Wastewater Treatment

**DOI:** 10.3390/ma12182858

**Published:** 2019-09-05

**Authors:** Po-Hsiang Chang, Wei-Teh Jiang, Binoy Sarkar, Wendong Wang, Zhaohui Li

**Affiliations:** 1 School of Human Settlements and Civil Engineering, Xi’an Jiaotong University, 28 Xianning West Road, Xi’an 710049, China; 2 Department of Earth Sciences, National Cheng Kung University, 1 University Road, Tainan 70101, Taiwan; 3 Department of Animal and Plant Sciences, The University of Sheffield, Sheffield S10 2TN, UK; 4 Department of Geosciences, University of Wisconsin – Parkside, 900 Wood Road, Kenosha, WI 53144, USA

**Keywords:** β-receptor blocker drugs, clay minerals, cation exchange, hydrogen bonding, exfoliation, pharmaceutical wastewater treatment

## Abstract

The adsorption of atenolol (AT) from aqueous solutions by Ca-montmorillonite (SAz-2) was investigated in batch studies under different physicochemical conditions. The AT existed in neutral un-dissociated form at pH 10, and was adsorbed on dioctahedral smectite (SAz-2) obeying the Langmuir isotherm with a maximum adsorption capacity of 330 mmol/kg. The kinetic adsorption suggested that both strong and weak adsorption sites existed on SAz-2 and participated in the adsorption mechanisms. The amount of exchangeable cations desorbed from SAz-2 during AT adsorption was linearly correlated with the amounts of adsorbed AT having slopes of 0.43, which implied that a cation exchange based adsorption mechanism was also in place. A comprehensive basal spacing change of SAz-2 was observed after AT adsorption on the clay mineral when tested with or without AT recrystallization. The intercalation of AT into the SAz-2 interlayers did not result in swelling due to the low adsorption capacity of the drug. Prominent interactions between the pharmaceutical molecule and SAz-2 were evidenced by apparent shifts of the infrared absorption bands after adsorption. The interlayer configurations and hydrogen bonding of AT on SAz-2 were also supported by infrared, X-ray diffraction and thermogravimetric analyses. This study suggested that SAz-2 is an excellent material to remove not only AT from pharmaceutical wastewater, but can potentially remove many other β-receptor blocker drugs. The results helped us to understand the possible interlayer configurations and adsorption mechanisms of the drugs on natural clay mineral based adsorbents.

## 1. Introduction

Atenolol (AT) is one kind of the β-receptor blocking agents, and is commonly prescribed for the therapy of hypertension, angina, arrhythmia, glaucoma, and against coronary heart diseases [1]. Like other anti-hypertension drugs, AT lowers the systolic and diastolic blood pressure by 15 to 20% in a single drug treatment, but is incompletely absorbed (50% of the drug is bioavailable) in the human body, and hence enter into the environment following human excretion [2].

Detection of β-blockers in wastewater, surface water, and groundwater suggested their incomplete removal in wastewater treatment plants (WWTPs) [3,4,5,6,7]. The AT is also released into the environment through urban wastewater treatment plant discharges [8], and the levels detected are similar to each other in WWTPs in Spain, Germany, Italy, and USA [9,10,11,12,13]. In the raw effluents of a Spanish WWTP, the corresponding concentration values were 0.84–2.8 μg/L for AT. Meanwhile, the daily aqueous mass output loads of AT in the treated wastewater were as high as 2.2–50.8 g/day [14]. Furthermore, the removal rate was only 69% with an influent concentration of 1.3 μg/L at a sewage treatment plant in Germany [15]. The measured concentrations of AT were 1.5–2.6 μg/L in raw sewages in Switzerland [16]. Ten surface water samples collected from the Ebro river basin in Spain showed the highest detected AT concentration of 250 ng/L (average 72 ng/L) [8]. Like other medical pollutants, the detection of AT in effluents of WWTPs therefore indicated difficulties of their removal from the environment.

Due to their high adsorption capacity and low price, clay minerals and natural zeolites were studied as adsorbents for water treatment [17,18]. Specifically, AT adsorption on modified-zeolite [19,20], modified-clinoptilolite, natural and modified clays such as kaolin and bentonite [17] were studied. However, there is still a continuous need to find inexpensive solid adsorbents that can fulfill high AT removal efficiency. On the other hand, in most previous studies, the mechanisms of drug adsorption on clay minerals were often investigated under neutral conditions without pH adjustment, or the adsorbate existed in its cationic form in favor of the ion exchange reaction [18,21,22]. One of the greatest challenges in our understanding of the adsorption of pharmaceutical contaminants is to characterize the mechanism under acidic or alkaline conditions because these conditions are frequently encountered in the natural aquatic environment. In this study, we tested AT adsorption on Ca-montmorillonite to discuss the adsorption mechanisms and interlayer configurations of the clay mineral under alkaline conditions without pH adjustment. The results could help us to predict possible mechanisms of other β-blockers during their adsorption on natural Earth materials under alkaline conditions.

## 2. Materials and Methods 

### 2.1. Materials

**Figure 1:**
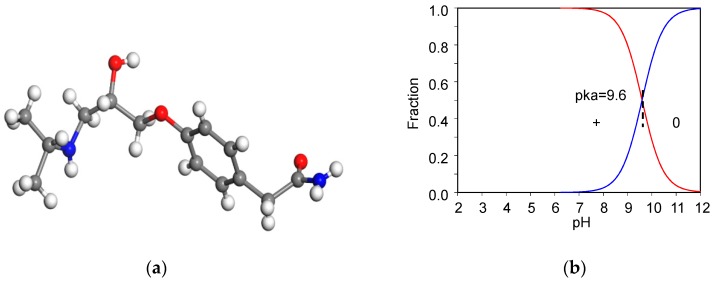
Molecular structure of AT (**a**) and their speciation under different pH values (**b**), respectively.

The 99.9% AT powder was provided by Swiss Pharmaceutical Co., Ltd, Tainan, Taiwan. AT has a molecular weight of 266 g/mol [8]. The chemical formula for AT is C_14_H_22_N_2_O_3_ (Figure 1a). The acid dissociation constant (pKa) of AT is 9.6 (Figure 1b) [23], the melting point is 152 °C [24], and the aqueous solubility (log(1/S_0_)) of AT is 1.72 [25]. The dynamic volume for AT is 343 Å^3^ [26]. The β-blocker could behave as a weak cationic surfactant at neutral pH [27].

The clay mineral used in this study was a dioctahedral smectite (SAz-2), obtained from the Clay Minerals Society Source Clay Repository. The sample received was in a chunky form, and was crushed to powder before being used. However, further purification was not performed. It contained 95%–100% montmorillonite, and 1%–2% quartz with a chemical composition of 60.4% SiO_2_, 17.6% Al_2_O_3_, 0.24% TiO_2_, 1.42% Fe_2_O_3_, 0.10% FeO, 0.08% MnO, 6.46% MgO, 2.82% CaO, 0.06% Na_2_O, 0.19% K_2_O, 0.02% P_2_O_5_, and 0.29% F in weight. The clay mineral’s reported cation exchange capacity (CEC) and specific surface area (SSA) values were 1.2 meq/g and 97 m^2^/g, respectively, with Ca as the major exchangeable cation [21].

### 2.2. Batch AT Adsorption Experiments

To each 50 mL centrifuge tube, 0.1 g of SAz-2 and 20 mL of AT solution were combined. For the isotherm study, the ambient pH of AT stock solution was 10.15, while the final pH values after adsorption were 8.54, 9.05, 9.42, 9.58 under initial AT concentrations of 25, 50, 100, 200 mg/L, respectively. However, the final pH values above *pKa* 9.6 for initial AT concentrations of 400–9000 mg/L did not change after the adsorption experiment. The pH of the raw SAz-2 suspension (as a control) was 8.3. All tubes were wrapped with aluminum foils to prevent light-induced decomposition of AT. The stock solutions were newly prepared for each batch to avoid the degradation. For the kinetic study, the mixing time was 0.25, 0.5, 1.0, 1.3, 1.6, 2.0, 4.0, 8.0, 10.0, 12.0, 14.0, 16.0, and 24.0 h under initial AT concentration of 800 mg/L corresponding to 3 mmol/L, while they were 0.25, 0.5, 1.0, 2.0, 4.0, 8.0, 16.0, and 24.0 h under initial AT concentration of 2000 mg/L corresponding to 7.5 mmol/L. For other experiments, the initial AT concentration were 3 mmol/L. For pH adsorption edge experiment, the equilibrium solution pH was varied between 2 and 11, and was adjusted by adding 2 mol/L NaOH or 2 mol/L HCl drop-wise, and pH was checked periodically. The purpose of using high concentrations of NaOH or HCl was to minimize the change in total liquid volume. For the ionic strength experiment, NaCl was used as the ionic strength adjustor with concentrations of 0.01, 0.05, 0.1, 0.5, and 1.0 mol/L. For temperature-dependent adsorption tests, the temperature was maintained at 298, 318, and 333 K. The mixtures were shaken on a reciprocal shaker at 150 rpm for 24 h for all experiments excluding the kinetic studies. After equilibration, the mixtures were centrifuged at 5000 rpm for 5 min, and the supernatants were passed through 0.22-um filters before being analyzed by an UV-visible (UV-VIS) spectrometer (SmartSpec 3000, Bio-Rad Corp., San Diego, CA, USA) and ion chromatography (Dionex 100, Dionex, Sunnyvale, CA, USA). All experiments were run in duplicate, and average values were reported. Although the concentrations of AT used in this study were much higher than the real environmental concentrations, the goal of this study was mainly focused on the mechanisms and interlayer configurations of AT adsorption on SAz-2. Furthermore, the presupposed evidence for the exfoliation of SAz-2 during reacting with AT was also discussed.

### 2.3. Methods of Analyses

All pH measurements were done using an Accumet™ AE150 pH Benchtop Meters (Thermo Fisher Scientific, Waltham, MA, USA). The AT was quantified by a SmartSpec 3000 UV-VIS spectrometer (Bio-Rad Corp., San Diego, CA, USA) at the wavelength of 273 nm [28]. The standards were adjusted to the same pH as the supernatants. A calibration curve was made with five standards between 10 and 100 mg/L with *r*^2^ value no less than 0.998.

The metal cations desorbed from the clay mineral during the adsorption of AT were analyzed by ion chromatography (Dionex 100, Dionex, Sunnyvale, CA, USA) with an IonPac Cs12A column (4 mm × 250 mm) and a mobile phase made of 1.922 mL of 20 mM methanesulfonic acid in 1 L of water. At a flow rate of 1 mL/min, the retention time for Na^+^, K^+^, Mg^2+^, and Ca^2+^ was 3.2, 4.4, 5.2, and 6.0 min, respectively.

Powder X-ray diffraction (XRD) patterns of samples were recorded on a Bruker D8 Advance Diffractometer (Bruker, Hamburg, Germany) equipped with a Sol-X detector, using Cu-kα1 radiation in the 2°–20° 2θ range and at a counting rate of 0.01 s/step. 

Thermogravimetric (TG) analyses were performed on a Perkin Elmer TGA4000 instrument (DKSH Technology, Toa Payoh, Singapore) with a heating rate of 10 °C/min under N_2_ condition. The initial sample weight was between 5 and 7 mg.

Fourier transform infrared (FTIR) spectra were acquired on a Thermo Nicolet 6700 spectrometer (Thermo Scientific, Waltham, MA, USA) using the KBr pressing method. The spectra were obtained from 400 to 4000 cm^–1^ by accumulating 256 scans at a resolution of 4 cm^−1^. 

## 3. Results and Discussion

### 3.1. Batch Studies

#### 3.1.1. AT Adsorption Kinetics

**Figure 2:**
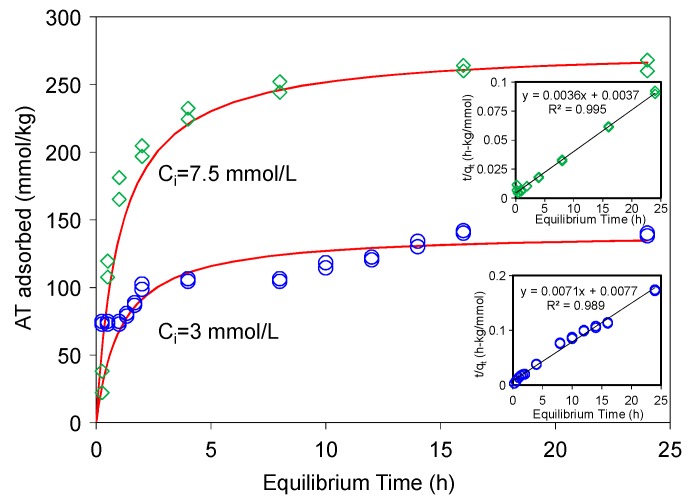
Sorption kinetics of AT on SAz-2 at pH10 under initial concentrations of 3 and 7.5 mmol/L, respectively. The solid line is pseudo-second-order model fitting of the observed data. Inserts are the linear plots of Equation (1).

AT adsorption on a modified-zeolite [17] showed that 24 h was needed to reach the equilibrium. Thus, the kinetic study was conducted first to determine the time required for attaining AT adsorption equilibrium on the SAz-2. The adsorption reached equilibrium in about 16 h for AT (Figure 2). Several kinetic models were used to fit the experimental data, and the pseudo-second-order kinetic model achieved the best fitting results. This model has been used to describe chemisorption of adsorbates and widely applied to the adsorption of pollutants from aqueous solutions in recent years. The integrated rate law for this model is Equation (1) [29,30]:

(1) $$q_{t}=\frac{kq_{e}^{2}t}{1+kq_{e}t}$$

where, *k* (kg/mmol-h) is the rate constant of adsorption, *q_e_* (mmol/kg) is the amount of AT adsorbed at equilibrium, and *q_t_* (mmol/kg) is the amount of AT adsorbed on the surface of the adsorbent at any time, *t*. The Equation (1) based on the adsorption capacity is proportional to the number of active sites occupied on the adsorbent, and can be re-arranged into a linear form (Equation (2)):

(2) $$\frac{t}{q_{t}}=\frac{1}{kq_{e}^{2}}+\frac{1}{q_{e}}t$$

where, *kq_e_*^2^ is the initial rate (mmol/kg·h). For AT adsorption at initial concentrations of 3 and 7.5 mmol/L, the initial rates were 129 and 270 mmol/(kg·h), the rate constants were 0.007 and 0.004 kg/(mmol·h), and the *q_e_* were 140 and 277 mmol/kg, when the kinetic data were fitted to Equation (2) (Figure 2), with the coefficients of determination values (*R*^2^) being 0.989 and 0.995, respectively. The pseudo-second-order kinetic model is based on the assumption that the rate-limiting step may be chemical sorption or chemisorption involving valence forces through sharing or exchange of electrons between the adsorbent and adsorbate, and this model provided the best correlation of the experimental data in this study [30,31]. 

Such a high initial rate and rate constant indicated that SAz-2 could be an outstanding adsorbent for AT. There was an interesting phenomenon that the gradual increase of adsorbed amounts of AT differed from 1 h to 2 h, and 8 h to 16 h for the initial AT concentration of 3 mmol/L (Figure 2). This adsorption behavior suggested two different adsorption sites that were possibly the strong and weak sites respectively [32]. In the beginning 1 h, the adsorption capacity was 75 mmol/kg (54% of total adsorption capacity), which could be from a speedy monolayer adsorption at the strong sites, after which the equilibrium process might be originated from a multilayer adsorption on weak adsorption sites [32]. Most of the kinetic adsorption models showed gradual increases of sorption due to the main mechanism of cation exchange [18,21,22,30,31]. However, results from this study showed that the adsorption did not change from 2 to 8 h at the low concentration of 3 mmol/L (Figure 2). We deduced that mechanisms other than cation exchange might exist. In the future, we need to make sure the real condition for kinetic process via the different initial concentrations of adsorbates to obtain more information to discuss the adsorption mechanism between clays and drugs.

#### 3.1.2. AT Equilibrium Adsorption

**Figure 3:**
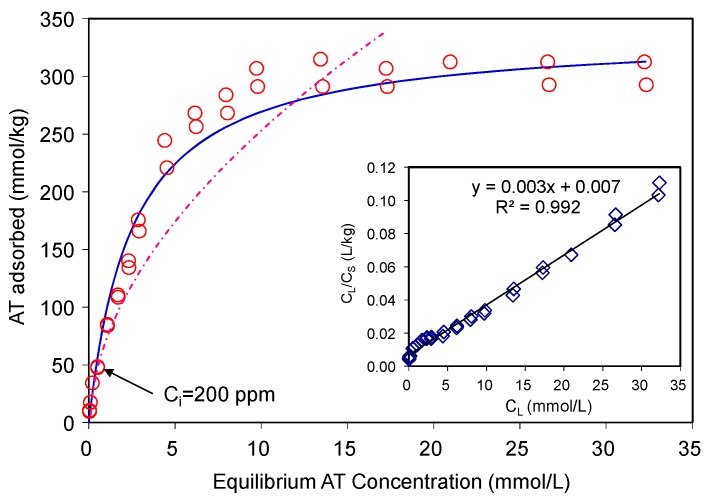
Adsorption of atenolol on SAz-2 at pH10. The dashed line is the Freundlich model fitting, the solid line is the Langmuir model fitting, which align to the observed data.

The AT adsorption data were fitted using the Langmuir and Freundlich isotherm models (Figure 3). The Langmuir equation can be described as Equation (3):

(3) $$C_{S}=\frac{K_{L}S_{m}C_{\cdots }}{1+K_{L}C_{\cdots }}$$

where, *C_S_* is the amount of adsorbate adsorbed on solid at equilibrium (mmol/kg), *C_L_* is the equilibrium solute concentration (mmol/L), *S_m_* is the apparent adsorption capacity or adsorption maximum (mmol/kg), and *K_L_* is the Langmuir coefficient (L/mg). The Equation (3) can be rearranged to a linear form (Equation (4)) so that *K_L_* and *S_m_* can be determined by a linear regression:

(4) $$\frac{C_{L}}{C_{S}}=\frac{1}{K_{L}S_{m}}+\frac{C_{L}}{S_{m}}$$

The Freundlich adsorption equation can be written as (Equation (5)):

(5) $$C_{s}=K_{F}C_{L}{}^{1/n}$$

where, *K_F_* is the Freundlich adsorption constant, and *n* is the Freundlich exponent. For AT, the *R^2^* value of the Langmuir fitting was (0.992) higher than that of the Freundlich fitting (0.950). The maximum AT adsorption capacity of the SAz-2 was 330 mmol/kg in comparison to 40, 25, and 25 mmol/kg AT adsorbed on natural zeolitic tuff, bentonite (CEC~0.922 meq/g) and kaolin (CEC~0.06 meq/g), respectively [17]. A modified zeolite, which had six different pore size distributions, resulted in AT adsorption to be tune of 320 mmol/kg [19] with corresponding adsorption capacity close to 176 mg/g [20]. On the other hand, the Freundlich isotherm model which involves multilayer adsorption on adsorbent surface fitted the experimental data poorly, with an R^2^ value of 0.95, and therefore was not adopted in this study. Thus, the Langmuir model was the best fitting model (Figure 3). This model is based on the assumption of monolayer adsorption and the adsorption capacity is limited by the number of active sites available on the adsorbent surface [18,21].

Although the results of adsorption capacity on various natural or modified clay minerals and other materials fail to be compared directly with the results reported here (because of totally different experimental conditions), it is still noteworthy to state that the effectiveness of SAz-2 for the removal of AT was remarkable. Compared to previous studies, the increased adsorption capacities obtained in this study imply that SAz-2 is an excellent candidate material to remove β-blocker drugs from aqueous media.

#### 3.1.3. Desorbed Cations

**Figure 4:**
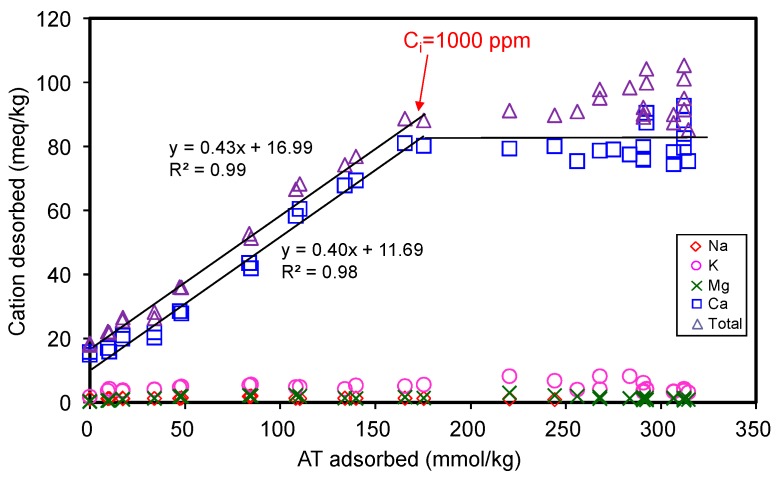
Desorption of metal cations from SAz-2 as affected by the amount of AT adsorption.

The total amount of cations desorbed during AT adsorption corresponded to 0.43 times that of AT adsorbed (Figure 4), suggesting only 43% of the total AT adsorption occurred via cation exchange mechanism. Thus, the major adsorption mechanism could not be attributed to the cation exchange phenomenon. Reasonably, the rest of AT adsorption amounts should be attributed to other mechanisms. The adsorption capacity of 330 mmol/kg for AT only accounted for 0.26 CEC of SAz-2, suggesting many of the exchangeable sites were not occupied by the AT molecules when the adsorption reached the equilibrium. The phenomenon could be due to the fact that the affinity between SAz-2 and AT was not strong due to the hydrophilic property of the drug molecules [28], or due to the dominance of its neutral (undissociated) form in the medium. Based on the kinetic study results, our inclination to the additional adsorption mechanisms therefore included the strong and weak adsorption sites theory at pH 10 for AT adsorption on SAz-2. Another possibility could be the multi-layer adsorption depending on the ratio of 0.43 between desorbed cations and adsorbed AT, and occupied area of AT on exchanged sites. Both these mechanisms are discussed in detail later in the manuscript.

#### 3.1.4. Effect of Solution pH and Ionic Strength on AT Adsorption

**Figure 5:**
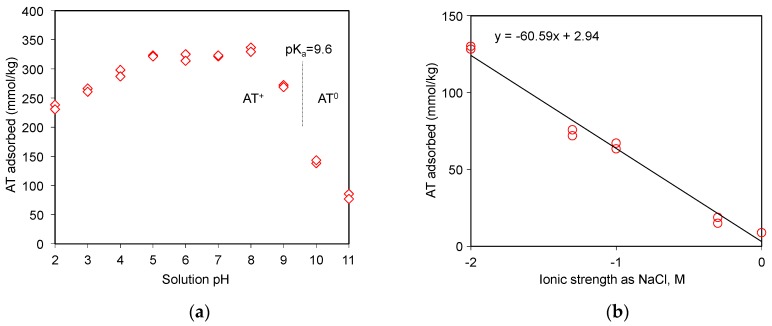
AT uptake on SAz-2 as affected by equilibrium solution pH (**a**) and ionic strengths (**b**) at an initial AT concentration of 3 mmol/L.

The role of electrostatic interaction between drugs and adsorbents could be investigated via the pH adsorption edge tests [20,21,33]. In fact, AT was a free base drug at pH 10.2 of the stock solution. The H^+^ ions could affect or compete against AT for the adsorption sites on SAz-2 under moderate to low pH (Figure 5a). The cationic form of AT could occur when the equilibrium solution pH was below the pKa of AT, and the adsorption amounts varied from 230 to 335 mmol/kg at pH 2 to pH 8 at an initial AT concentration of 3 mmol/L (Figure 5a). Above the pKa value, however, the AT adsorption as neutral molecules decreased abruptly to 145 mmol/kg at pH 10, and further low to 80 mmol/kg at pH 11. The adsorption was therefore driven solely by hydrophobic interactions instead of electrostatic interactions under alkaline conditions [20]. 

As the ionic strength of NaCl solutions increased from 0.01 to 1.0 mol/L, the adsorbed amounts of AT decreased from 130 to 9 mmol/kg at an initial concentration of 3 mmol/L (Figure 5b), suggesting that Na^+^ competed against AT for the exchangeable adsorption sites of SAz-2. On the other hand, the hydrophobic interaction between the drug molecules might overcome the repulsive electrostatic interaction, which favored the aggregation of clay particles at high ionic strength, and further influenced the adsorption capacity [34]. Similar results were observed for AT adsorption on beta zeolite [20]. Even though the cation exchange was not the main adsorption mechanism for AT at pH 10, the slope of 0.43 (Figure 4) suggested a significant cation exchange contribution to AT adsorption on SAz-2. Therefore, the competition from Na^+^ still influenced the AT adsorption capacity on SAz-2 at high pH values. 

On the other hand, although β-blockers are known as weak cationic surfactants [27], and are capable of forming micelles through their weak surface activity [35], it does not always imply that adsorption of these molecules would be as strong as that produced by true surfactants. For example, the adsorption of the cationic surfactant cetylpyridinium chloride (CPC) to 80–140 mesh Canadian River alluvium [36] exhibited a K_D_ value of approximately 1300 mL/g at low concentrations, which was two orders of magnitude higher than propranolol. Similarly, the results of this study also revealed this contention.

#### 3.1.5. Influence of Temperature on AT Uptake on SAz-2

**Figure 6:**
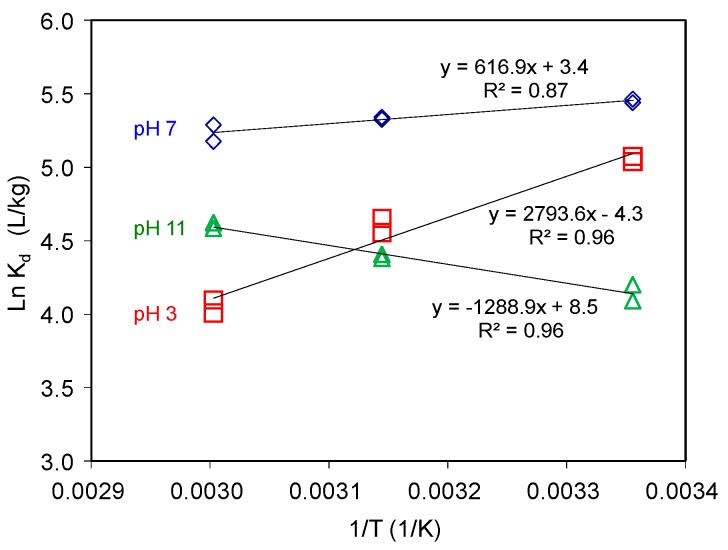
AT adsorption on SAz-2 as affected by equilibrium temperature at pH 3, 7 and 10 under initial AT concentration of 3 mmol/L.

At an initial concentration of 3 mmol/L, under neutral condition, the AT adsorption capacities decreased with increasing temperature (Figure 6), suggesting an exothermic adsorption process. On the contrary, under the alkaline condition, the adsorption capacities increased with increasing temperature, suggesting an endothermic adsorption process. The thermodynamic parameters of AT adsorption are related to the partitioning coefficient as in Equation (6):

(6) $$\ln K_{d}=-\frac{\Delta H}{RT}+\frac{\Delta S}{R}$$

where, *T* is the temperature in K, *R* is the gas constant, *ΔH* is the change in enthalpy, and *ΔS* is the change in entropy after adsorption. The free energy of adsorption *ΔG* is linked to these thermodynamic parameters by (Equation (7)):

(7) ΔG=ΔH−TΔS

The small negative *ΔG* values suggested physical adsorption (Table 1). Generally, the permutation of adsorbed adsorbate on the adsorbent surface would change from disordered to ordered arrangement with the adsorption capacity gradually reaching to the CEC value of adsorbent under cation exchange mechanism [21,22]. Since the adsorption reached to equilibrium, we could elaborate these implications via the thermodynamic parameters [22,37]. However, the adsorption capacities of AT on SAz-2 were much less than the CEC value of SAz-2. As a result, the AT molecules could be arranged randomly, their ordered or disordered morphologies were incomparable with the adsorption capacities in this study.

### 3.2. XRD Analyses

**Figure 7:**
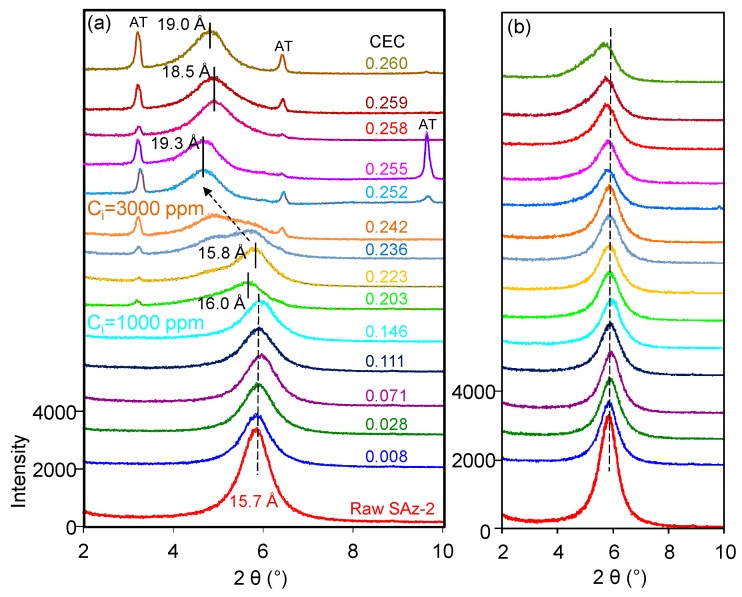
XRD patterns of raw SAz-2 and SAz-2 with different amounts of AT intercalation at pH10 when XRD thin sections were made with (**a**) and without (**b**) AT recrystallization, respectively.

The *d_001_* value of the raw SAz-2 was 15.7 Å (Figure 7a) compared to 15.5 Å for SAz-1 [22] confirming that the interlayer cation was Ca^2+^ associated with two layers of water. The d-value remained the same after SAz-2 was equilibrated with AT below an initial concentration of 1000 mg/L corresponding to 3.75 mmol/L (Figure 7a). In a previous study [21], the SAz-2 showed a gradual swelling of the interlayers with adsorption capacities of amitriptyline increased. However, the irregular change of d-value in this study implied that the arrangement morphologies of AT molecules in the SAz-2 interlayers were incomplete. We inadvertently found the possible exfoliation of SAz-2 by virtue of recrystallization of AT especially at high initial adsorbate concentrations (Figure 7a). To confirm this, we dropped 1 to 1.5 mL of SAz-2-AT suspension on a XRD slide forming thin section samples and naturally dried under ambient environment. Thus, the recrystallization of AT appeared on the patterns due to the high concentration of residual AT in the adsorbed liquid samples (Figure 7a). Therefore, we made thin sections again excluding the influence of recrystallization (Supplementary Materials). We found that the changes of *d_001_* values were consistent as before (Figure 7b), but the degree of peak shifts were different to that shown in Figure 7a. However, the non-swelling behavior of SAz-2 did not mean that AT did not intercalate in SAz-2. For example, the d-value of samples changed to 13.5 Å, but it change to 9.7 Å for the raw SAz-2 when heated at 400 °C (Figure S1c). Consequently, the variation of d-values supported the intercalation of AT under four heating temperatures (Figure S1a–d).

### 3.3. Thermogravimetric (TG) Analyses and Possible Exfoliation

**Figure 8:**
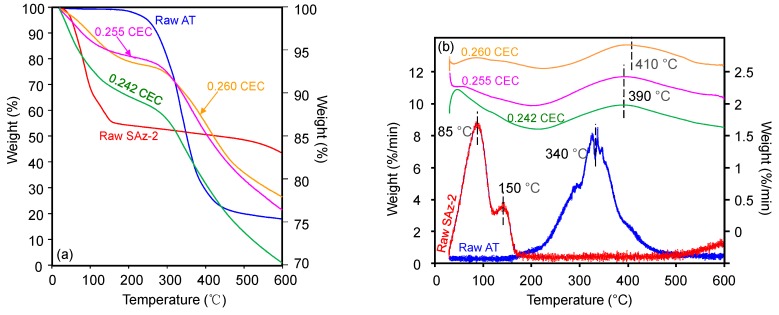
TG analyses of raw SAz-2, and SAz-2 with AT adsorbed amounts equivalent to 0.242, 0.255 and 0.260 CEC of the clay mineral (**a**); the vertical scale of figure for raw AT was on the left side, for the others were on the right side. Also drawn is the DTG analyses of them (**b**); the vertical scale of raw SAz-2 and SAz-2-AT were on the right side, and raw-AT was on the left side.

**Figure 9:**
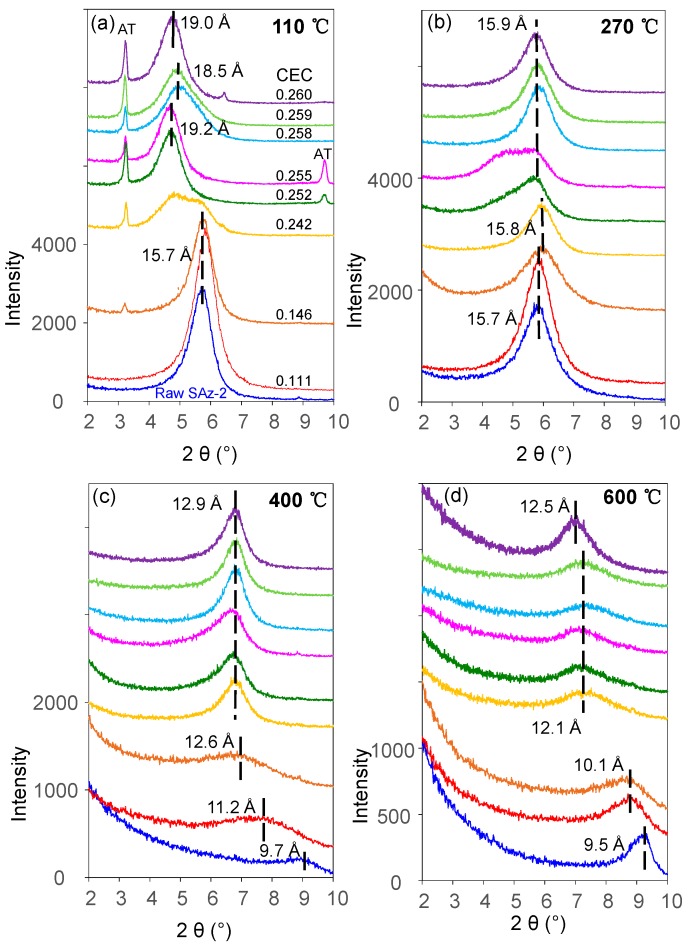
XRD patterns of raw SAz-2 and SAz-2 with different adsorbed amounts of AT without recrystallization from isotherm adsorption for AT under four different heating temperatures: (**a**) 110 °C; (**b**) 270 °C; (**c**) 400 °C; (**d**) 600 °C.

The TG (Figure 8a) and derivative of TG (DTG) (Figure 8b) curves of raw AT showed a peak decomposition temperature (Tpeak) of 340 °C with a mass loss of 80% continuing up to 500 °C (Figure 8a). Although the mass loss was close to 20%, the Tpeak at 150 °C for AT was still indicating the melting point of AT (Figure 8a) [24]. The TG analysis result of AT was in agreement with those of previous studies which reported a mass loss of 74.6% from 201 °C up to 507 °C [37]. Besides, the previous study on SAz-2 showed that the Tpeaks of 85 °C and 145°C (Figure 8b) were attributed to the removal of adsorbed water and interlayer water, respectively [38]. The Tpeak increased to 390 °C and 410 °C for AT-loaded SAz-2 samples with AT loadings equivalent to 0.255 and 0.260 CEC of the clay mineral (Figure 8b), respectively, which indicated the intercalation phenomenon [39]. Furthermore, if the adsorption occurred only on the surface instead of intercalation, the d-values of the AT-loaded clay mineral and the raw clay sample should be consistent. In the current study, the change of d001 values was not consistent when the At-loaded SAz-2 was heated at 400 and 600 °C (Figure 9). Therefore, we deduced that intercalation indeed occurred. Moreover, the unclear peaks at Tpeaks of 390 °C and 410 °C not only suggested the adsorption capacities were small, but also agreed with the results of the adsorption isotherm study. The DTG results not only confirmed that the AT molecules replaced the hydrated Ca^2+^ ions in the interlayers [33], but also supported that the decomposition of AT from the internal surface of SAz-2 occurred in addition to the external surface of the clay mineral [40]. 

The fluctuation of d_001_ values with the adsorbed amounts of AT was apparently inconclusive in this study (Figure 7a). However, the same effect was confirmed with additional heat treatment experiments of the AT-loaded SAz-2 samples. When heated at 110 °C (Figure 9a), we found the d001 value changed to a mix-layer condition compared to the original one at 0.242 CEC. When the temperature was increased to 270 °C (Figure 9b), the d001 value changed to a mix-layer condition compared to the loading in the interlayer at 0.255 CEC. The AT residues totally disappeared because the decomposition temperature of AT was about 200 °C (Figure 8b). The d001 value change from one broad peak to a mix-layer could be influenced by the recrystallization of AT. In the beginning, we need to drop about 1 ml of the mixture suspension of SAz-2 and AT on the thin section to make the XRD sample to determine the d-spacing of them after ambient drying. Since we unremoved the highly residual concentration of AT during XRD thin section preparation, so the AT should recrystallize after drying and the crystals should be land on the outer surface rather than the interlayers of SAz-2. However, the result doesn’t support this assumption (Figure 9a,b). Since the exfoliation does happen, the AT crystals could possible land on the inner surface of clay sheets after ambient drying. Furthermore, the interlayer adsorbed AT molecules would be together with them after sheets structure were rebuild. If this hypothesis was correct, the sheets of SAz-2 could exfoliate in the solution and re-bind after drying following adsorption. On the other hand, when the AT crystals disappeared, it did not influence the d001 values, which were consistent at 12.9 and 12.5 Å at the temperature of 400 and 600 °C, respectively (Figure 9c,d). Therefore, these AT crystals played a significant role in the irregular changes of d001 values. Although we found the phenomenon of exfoliation by indirect evidence here, we need to further study to confirm these results, possibly with other compounds that have recrystallization property during adsorption on SAz-2. In depth scanning electron microscopy (SEM), transmission electron microscopy (TEM), and X-ray photoelectron spectroscopy (XPS) investigations would be useful to directly obtain exfoliation evidence.

### 3.4. FTIR Analyses

**Figure 10:**
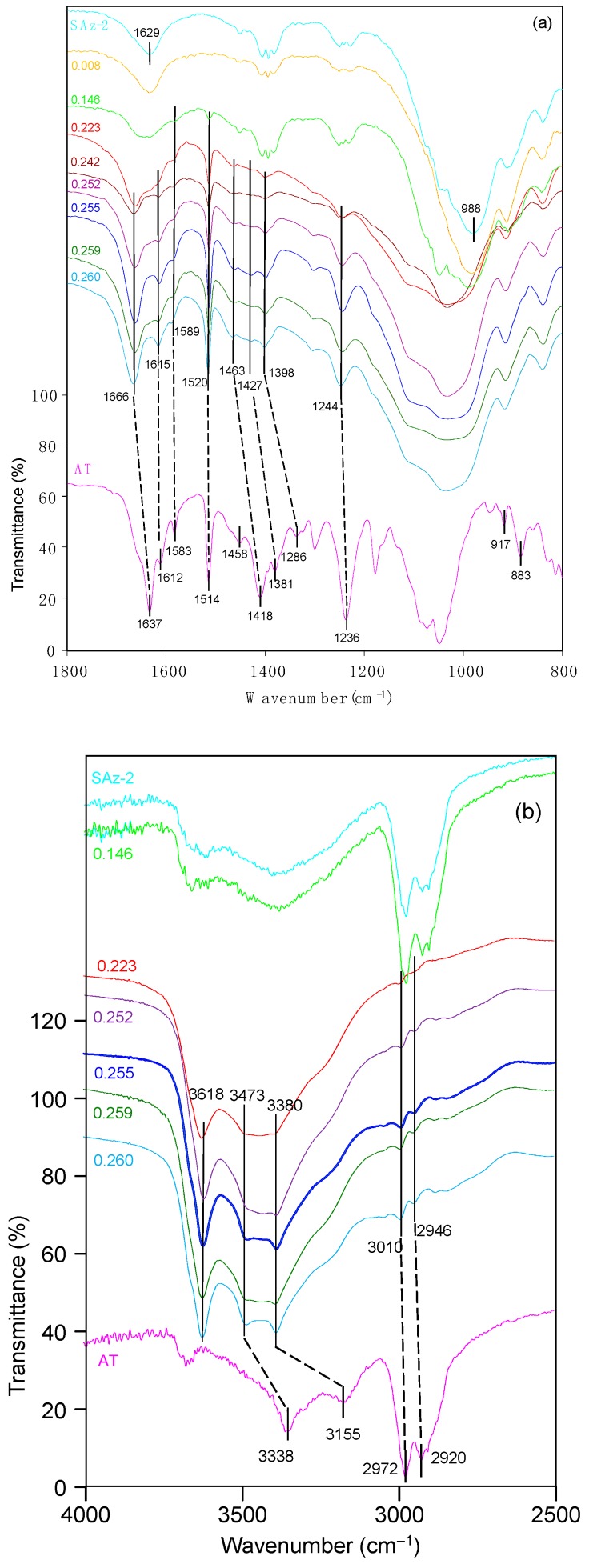
(**a**) FTIR spectra of raw SAz-2, solid AT, and SAz-2 with different amounts of AT adsorption. The values of left side are in CEC. The range is 800−1800 cm^−1^. **(b)** FTIR spectra of raw SAz-2, solid AT, and SAz-2 with different amounts of AT adsorption. The values of left side are in CEC. The range is 2500–4000 cm^−1^.

The related band positions were listed in Table 2. As for AT, the 3338 cm^−1^ band corresponded to NH bending vibration, and the peak at 3155 cm^−1^ was due to N-H valence vibration [40]. The band at 2972 cm^−1^ was due to C–H stretching vibration. The strong ν(C=O) band vibrations at 1637 cm^−1^ was shifted to a higher frequency at 1666 cm^−1^ following AT adsorption at 0.14 CEC of the clay mineral (Figure 10a), indicating the involvement of this oxygen atom in the coordination reaction [41]. The ring stretching frequencies appeared at 1612 and 1583 cm^−1^ were clear in the adsorption complexes. Thus, it gave evidence for strong interaction of the aromatic ring of AT molecules with the clay mineral surfaces. The benzene ring skeletal vibration was observed at 1514 cm^−1^ which shifted to 1520 cm^−1^ after AT adsorption on SAz-2. The band position at 1418 cm^−1^ for NH bending vibrations shifted to 1453 cm^−1^ after AT adsorption. The band position at 1381 cm^−1^ shifted to 1427 cm^−1^, suggesting the interaction of the molecules with the SAz-2 surface through the COH groups. The peak at 1236 cm^−1^ was due to the alkyl aryl ether linkage which shifted to 1244 cm^−1^. All of these band shifts were of the blue shift patterns. Enhanced bands at 917 and 883 cm^−1^, corresponding to the δ(OH) and ρ(NH_2_) vibrations were overlapped by the prominent band at 908 cm^−1^ for SAz-2 (Figure 10a). These two vibrations were coupled with the deformation of H_3_C-CH-CH_3_ angle, and could not be identified in this study. Therefore, the adsorption was not dependent on atoms of the chain structure of AT, and indicated that the AT molecular chain experienced a slight tilting while adsorbing on the clay mineral surface [41]. Based on the analysis of the FTIR spectra, the adsorption mechanism of AT on SAz-2 could be deduced as hydrogen bonding via the benzene ring, COH groups and NH groups of the AT molecules.

## 4. Adsorption Mechanism

**Figure 11:**
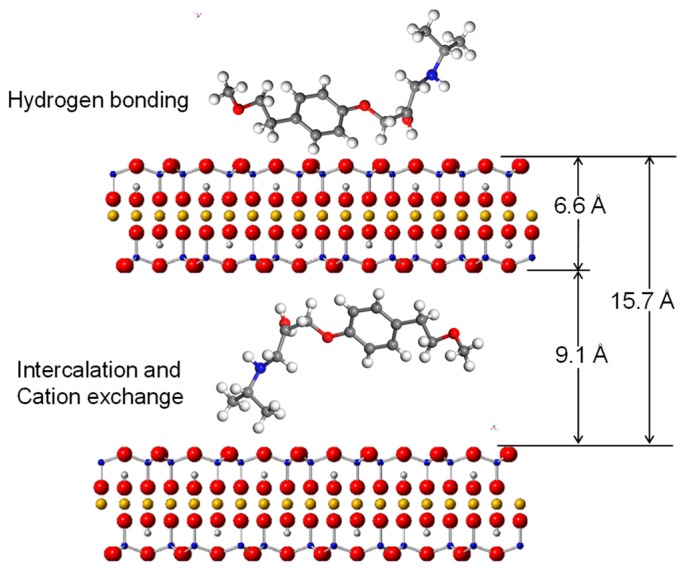
Illustration of intercalated AT inside the interlayers and in the outer surface of SAz-2 based on adsorption mechanism at low AT loading level.

The dimension of AT is 7 × 18.2 Å [43], while the dynamic volume is 343 Å^3^ [26]. Therefore, the theoretical molecular size is 7 × 18.2 × 2.7 Å for AT. As indicated by the XRD and FTIR results and the morphologies of the AT molecule, the drug molecules were likely inserted in a parallel style into the clay interlayers (Figure 11). The dimension of T-O-T layers of the 2:1 structure of the clay mineral is about 6.6 Å [44]. In this case, the molecular size of AT was enough to intercalate into the inter-lamellar space of SAz-2 (15.7 – 6.6 = 9.1 Å) by means of various configurations. This hypothesis further supported the AT intercalation theory in the SAz-2 interlayers (Figure 11). Interestingly, SAz-2 is an expandable clay mineral, but its basal spacing did not apparently increase after AT adsorption (Figure 8). This results were comparable with the adsorption of tetracycline [22], ciprofloxacin [44] and amitriptyline [21] on SAz-2. The SAz-2 has very high capacities to adsorb these drugs, and the external surface area was not able to accommodate all amounts of the adsorbed adsorbates.

The cation exchange was one of the adsorption mechanisms of AT on SAz-2 (Figure 4). The adsorption amounts gradually increased at the initial AT concentration of 1000 mg/L with simultaneous metal cation desorption, but reached the equilibrium at 3000 mg/L AT concentration without significant metal cation desorption. It was obvious that the neutral form AT could not totally exchange with metal cations on the surface of SAz-2. Moreover, according to the results of FTIR, the hydrogen bonding was confirmed as the second AT adsorption mechanism in this study. This kind of weak bonding resulted in the low adsorption capacity and slow adsorption rate of AT. Results of previous studies indicated that hydrophobicity was the only factor influencing the adsorption of AT on mineral surfaces [35]. Indeed, the affinity between AT and SAz-2 surfaces were not very high (as shown from the results of the effects of pH and ionic strength experiments), and the adsorption capacities were low in this study. On the other hand, the exfoliation of SAz-2 layers could happen. Since the layers of SAz-2 were separated in the suspension, the metal cations on the layer surfaces would partly be exchanged with AT, strengthening the cation exchange mechanism. Hydrogen bonding and adsorption on weak and strong adsorption sites remain the other possible AT adsorption mechanisms on SAz-2.

Nevertheless, our study pointed out that strong and weak adsorption sites, cation exchange and hydrogen bonding were the adsorption mechanisms for AT removal by SAz-2 at pH 10. Generally, we cannot determine the adsorption mechanism of one drug on an adsorbent at alkaline or acidic conditions due to the addition of H^+^ or OH^−^ ions. However, the results of this study enabled us to predict the adsorption mechanisms under alkaline conditions. Similarly, we can predict the adsorption mechanisms of other β-receptor blocker drugs such as metoprolol, practolol, pindolol, oxprenolol and alprenolol on clay minerals under alkaline conditions.

To further predict the interaction of other β-blocker drugs (e.g., metoprolol, practolol, pindolol, oxprenolol and alprenolol) with clay minerals at alkaline conditions, future investigations should include advanced microscopic and spectroscopic techniques such as SEM, TEM, XPS, etc. to confirm the interlayer exfoliation phenomenon directly.

## 5. Conclusions

From the results of this study the following conclusions can be drawn:

(1) The maximum AT adsorption on SAz-2 was 330 mmol/kg at pH 10. The adsorption data was well described by the Langmuir model. Cation exchange, hydrogen bonding and strong and weak adsorption sites were the mechanisms of AT removal by SAz-2 at pH 10.
(2) Adsorption of AT on SAz-2 was strongly dependent on solution pH and ionic strength.
(3) The basal spacing of SAz-2 remained unchanged after equilibrated with AT at different initial AT concentrations due to the lower adsorption capacity of the clay mineral. The AT intercalation without interlayer expansion was evidenced by a heating experiment with AT-adsorbed SAz-2 samples.
(4) The exfoliation of SAz-2 was indirectly confirmed based upon the basal spacing changes from ordered to mix-layers condition with increasing heating temperature of the samples at the same adsorption capacity. The recrystallization of AT played a significant role on the ordered or disordered conditions of *d_001_*.

## Figures and Tables

**Table 1:** Thermodynamic values of AT adsorption on SAz-2 under different temperatures.

	pH	Ln(K_d_) (L/Kg)			△G° (kJ/mol)			△H° (kJ/mol)	△S° (KJ/mol K)
		298 K	318 K	333 K	298 K	318 K	333 K		
AT	3	5.05	4.60	4.05	−12.62	−11.91	−11.37	−23.23	−0.04
	7	5.45	5.33	5.23	−13.51	−14.08	−14.50	−5.13	0.03
	10	4.14	4.39	4.60	−10.25	−11.66	−12.71	10.72	0.07

**Table 2:** FTIR band positions (cm^−1^) for crystalline AT and SAz-2 after adsorption at pH 10.

Crystalline SAz-2	Samples	Possible band assignment [42]
1629	1644	OH deformation of water
988	992	Si-O stretching
Crystalline AT	Samples	Possible band assignment [40]
3338	3473	NH bending vibration
3155	3380	N–H valence vibrations
2972	3010	C–H stretching
1637	1666	ν(CO)
1612	1615	ν(CC ring)
1583	1589	ν(CC ring)
1514	1520	ν(CC ring) + δ(CH ring) + δ(CH_2_)
1458		δ(CH_3_)
1418	1463	δ(COH) + ω(CH_2_)
1381	1427	δ(COH) + ω(CH_2_)
1286	1398	δ(OCNH_2_) +δ(CCC ring)
1236	1244	ω(CH_2_)
917		δ(OH)
883		ρ(NH_2_)

## Supplementary Materials

The following are available online at https://www.mdpi.com/1996-1944/12/18/2858/s1, Figure S1: XRD patterns of raw SAz-2 and SAz-2 with different adsorbed amounts without recrystallization from isotherm adsorption for AT under four different heating temperatures (a–d).
