# Supplementary material for: The Triple Mechanisms of Atenolol Adsorption on Ca-Montmorillonite: Implication in Pharmaceutical Wastewater Treatment
Source: Materials (Basel). 2019 Sep 5;12(18):2858. doi: 10.3390/ma12182858 (PMC6765981; doi:10.3390/ma12182858)
Supplement: Supplementary file 1 [file materials-12-02858-s001.pdf]

materials

MDPI

Supplementary Materials

The Triple Mechanisms of Atenolol Adsorption on Ca-Montmorillonite: Implication in Pharmaceutical Wastewater Treatment

Po-Hsiang Chang [1], Wei-Teh Jiang [2,*], Binoy Sarkar [3], Wendong Wang [1,*] and Zhaohui Li [4,*]

1 School of Human Settlements and Civil Engineering, Xi'an Jiaotong University, 28 Xianning West Road, Xi'an 710049, China; tectonicion@xjtu.edu.cn
2 Department of Earth Sciences, National Cheng Kung University, 1 University Road, Tainan 70101, Taiwan;
3 Department of Animal and Plant Sciences, The University of Sheffield, Sheffield S10 2TN, UK
4 Department of Geosciences, University of Wisconsin – Parkside, 900 Wood Road, Kenosha, WI 53144, USA
* Correspondence: atwtj@mail.ncku.edu.tw (W.-T.J.); wdwang@xjtu.edu.cn (W.W.); li@uwp.edu (Z.L.); Tel.: 1-262-595-2487 (Z.L.)

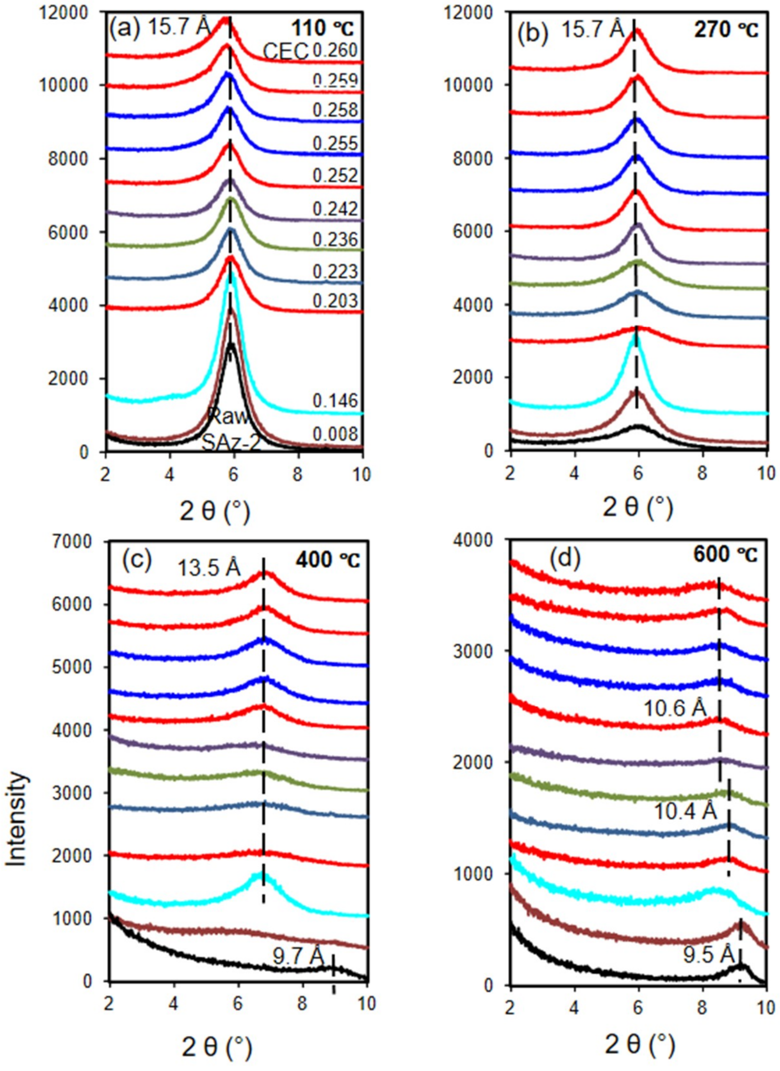

Figure S1. XRD patterns of raw SAz-2 and SAz-2 with different adsorbed amounts without recrystallization from isotherm adsorption for AT under four different heating temperatures (a–d).

© 2019 by the authors. Licensee MDPI, Basel, Switzerland. This article is an open access article distributed under the terms and conditions of the Creative Commons Attribution (CC BY) license (http://creativecommons.org/licenses/by/4.0/).
